# Early Linguistic Markers of Trauma-Specific Processing Predict Post-trauma Adjustment

**DOI:** 10.3389/fpsyt.2018.00645

**Published:** 2018-12-05

**Authors:** Birgit Kleim, Andrea B. Horn, Rainer Kraehenmann, Matthias R. Mehl, Anke Ehlers

**Affiliations:** ^1^Institute of Psychiatry, Psychology and Neuroscience, King's College London, London, United Kingdom; ^2^Department of Psychology, University of Zurich, Zurich, Switzerland; ^3^Department of Psychiatry, Psychotherapy and Psychosomatics, University of Zurich, Zurich, Switzerland; ^4^University Research Priority Program “Dynamics of Healthy Aging”, University of Zurich, Zurich, Switzerland; ^5^Department of Psychology, University of Arizona, Tucson, AZ, United States; ^6^Department of Experimental Psychology, Oxford University, Oxford, United Kingdom

**Keywords:** post-traumatic stress disorder, early predictors, cognitive processing, LIWC, text analysis, linguistic

## Abstract

Identifying early predictors for psychiatric disorders, such as post-traumatic stress disorder (PTSD), is crucial for effective treatment and prevention efforts. Obtaining such predictors is challenging and methodologically limited, for example by individuals' distress, arousal, and reduced introspective ability. We investigated the predictive power of language-based, implicit markers of psychological processes (*N* = 163) derived from computerized text-analysis of trauma and control narratives provided within 18 days post-trauma. Trauma narratives with fewer cognitive processing words (indicating less cognitive elaboration), more death-related words (indicating perceived threat to life), and more first-person singular pronouns (indicating self-immersed processing) predicted greater PTSD symptoms at 6 months. These effects were specific to trauma narratives and held after controlling for early PTSD symptom severity and verbal intelligence. When self-report questionnaires of related processes were considered together with the trauma narrative linguistic predictors, use of more first-person singular pronouns remained a significant predictor alongside self-reported mental defeat. Language-based processing markers may complement questionnaire measures in early forecasting of post-trauma adjustment.

## Early Linguistic Markers of Trauma-Specific Processing Predict Post-trauma Adjustment

There is large heterogeneity in psychological responding following exposure to traumatic events [e.g., (1)]. Trauma impact and recovery are not randomly distributed. Many survivors show a high degree of resilience and ultimately (and sometimes quickly) return to normal lives whilst others develop psychological disorders, such as post-traumatic stress disorder (PTSD). These survivors could benefit from professional help to mitigate the long-term social, emotional, and health impact of experiencing trauma (2). Fortunately, treatment options exist. Over the last decades, trauma researchers have made big strides in developing successful interventions and delivery of such interventions early after trauma has been shown to be effective (3).

A particularly important scientific question therefore is to identify early predictors of adjustment trajectories. To the extent that clinicians can reliably identify who is likely going to do well and who is at a high risk for developing chronic PTSD, limited therapeutic resources can be allocated to where they are most needed. In the context of trauma, identification of predictors at an early stage is an important objective. Survivors often have contact with professional services in the initial aftermath of a trauma, whereas such contact appears much more difficult to establish later, when disorders are chronic, often comorbid and more difficult to treat (4, 5).

Ideally, predictors of later chronic PTSD would be (a) early markers that can facilitate optimization of treatment initiation and resource allocation (b), naturally observable for clinicians—so that assessment burden can be kept to a necessary minimum at a distressing and vulnerable time for trauma survivors, and (c) trauma-specific—so that prediction errors are minimized. Finally, as the validity of self-report questionnaires may be undermined by factors such as high distress, emotional arousal, and limited introspective ability—factors that characterize information processing in the aftermath of a trauma- such markers would ideally be independent of the survivor's explicit self-report and could thus significantly complement such measures.

Natural language markers derived from individuals' spontaneous word use have recently received increased scientific attention (6). Use of certain words in individuals' writing or speech has been related to psychological aspects of their personal health and psychopathology [e.g. (7–10)]. Following trauma, maladaptive processing of traumatic memories may contribute to the development of PTSD. Linguistic features indexed in trauma survivors' personal accounts of their experience may offer more direct, “unfiltered” access to the way this experience is processed than self-report or interview measures. Unobtrusive indices of patterns of word use in such accounts may thus be good candidates to complement existing internal process measures for forecasting post-trauma adjustment [cf. (11, 12)]. Finally, linguistic measures reflect spontaneous behavior and thus do not share method variance with self-reported symptom outcomes, hence providing more unique and robust estimates of potential associations with PTSD symptoms.

Which trauma memory processing styles have been associated with later PTSD? According to cognitive models [e.g. (13, 14), survivors who engage primarily in surface-level processing of sensory and perceptual characteristics without elaboration of context and meaning of the event are more prone to develop PTSD than those who engage in more in-depth elaborate cognitive processing during trauma. Use of cognitive words in trauma narratives might reflect this elaboration process (15, 16). Peritraumatic mental defeat, a peritraumatic process, consisting of complete loss of inner resistance has also been implicated in the development in PTSD (14, 17, 18) as well as perceived threat to life (19). Earlier studies have indexed the use of death-related words in trauma narratives as linguistic indicator and proxy of these processes, which was related to PTSD as expected (20, 21). A meta-analysis of PTSD predictors highlighted survivors' emotional response to the trauma, such as fear, helplessness, horror, guilt, and shame, during trauma as one of the strongest PTSD predictors (19), a process captured by the use of negative emotion words in trauma narratives (22, 23). Finally, the use of first-person singular pronouns (“I,” “me.” “my”), a proposed measure of self-immersed processing (24, 25) has emerged as a predictor of depression (9, 26, 27) and general psychopathology (28) in prior research. Whilst working through the trauma memory is beneficial for constructing an elaborated and organized perspective of the event, a narrow and self-immersed perspective and focus on recounting details and personal reactions might undermine adaptive self-reflection (29) as well as the resolution of initial post-traumatic stress reactions.

Whereas, some studies have investigated language use following exposure to national traumatic events in the general population (30, 31) or in smaller samples of indirectly exposed individuals (32), no study has yet investigated whether such linguistic markers indexed in the early aftermath of trauma predict later chronic PTSD in a large sample of trauma survivors and how specific such linguistic predictors are in forecasting trauma adaptation and PTSD, over and above self-report questionnaires, as well as in complement to such self-report measures.

The present study investigated four candidate linguistic predictors, assessed early after assault, a trauma with increased PTSD risk relative to other potentially traumatic events (4). We indexed linguistic measures in trauma narratives provided on average 18 days post-trauma by a large sample of directly exposed trauma survivors. Knowledge about such early predictors is sparse, although it may help identify those survivors that could effectively be treated to prevent later chronic PTSD. In order to establish process-specificity of linguistic characteristics, we indexed linguistic markers in trauma narratives, as well as in non-traumatic control narratives. Specifically, we hypothesized that linguistic measures of (1) less elaboration and cognitive processing, (2) more mental defeat and threat to life, (3) more negative emotionality, and (4) more self-immersed processing in early accounts of the assault would be associated with more PTSD symptoms at 6 months follow-up. We then determined the extent to which significant trauma-specific linguistic predictors complement corresponding self-report questionnaires in predicting PTSD.

## Method

### Participants and Procedures

The local ethics board approved the study. Participants were assault survivors who attended the Emergency Department of a large urban teaching hospital. Exclusion criteria, assessed by an initial screening interview, were current psychosis, alcohol dependence, ongoing domestic violence, and no memory of the assault. Two hundred and twenty-two individuals were recruited and consented to participate in a research session conducted by a research psychologist, which took place around 18 days post-assault, *SD* = 9.7 days. During this session, 163 (67% male, mean age = 34 years, *SD* = 11.4) provided a narrative of their trauma and a negative, non-traumatic control event from around the same time of the assault. The latter was used to examine trauma-specificity of linguistic process measures^1^^,^^2^. Participants were asked to remember both events as vividly and in as much details as possible and to provide a detailed verbal report of their trauma including sensory impressions and cognitions during trauma. Narrative length was highly variable between participants, with mean narrative length of 786 words (*SD* = 584) for the trauma and 211 words (S*D* = 203) for the negative event narrative. Nearly all assaults were physical assaults; only 2% were sexual assaults. See Table 1 for descriptive statistics and correlations among study variables.

**Table 1:** Descriptive statistics and correlations among study variables.

		***M* (*SD*)**	**1**	**2**	**3**	**4**	**5**	**6**	**7**	**8**	**9**	**10**	**11**	**12**	**13**
1	Initial PTSD symptoms (PDS)	18.9 (12.4)	–												
2	PTSD symptoms 6 months (PSSI)	10.4 (10.0)	0.60^***^	–											
3	NART (number correct words)	23.9 (11.9)	−0.20^**^	−0.25^**^	–										
4	Cognitive processing NEN	18.0 (5.6)	0.15	−0.05	0.17^*^	–									
5	Cognitive processing TN	17.6 (3.3)	−0.16	−0.14	0.15	0.24^**^	–								
6	Negative emotions NEN	2.5 (1.8)	0.11	−0.06	0.08	0.18^*^	0.03	–							
7	Negative emotions TN	1.9 (1.0)	0.03	0.12	−0.06	0.01	0.11	0.09	–						
8	Death-related words NEN	0.05 (0.25)	−0.02	0.04	−0.05	−0.07	−0.04	0.05	0.07	–					
9	Death-related words TN	0.03 (0.09)	0.21^**^	0.27^**^	0.00	−0.01	0.01	−0.04	0.18^*^	0.12	–				
10	First person singular pronouns NEN	9.7 (3.7)	0.23^**^	0.14	−0.12	0.35^***^	−0.09	0.04	0.00	0.04	0.07	–			
11	First person singular pronouns TN	10.4 (2.7)	0.24^**^	0.29^**^	0.25^**^	−0.03	−0.02	0.10	0.06	−0.01	0.11	0.33^***^	–		
12	Data-driven processing (TPQ)	1.91 (1.05)	0.59^***^	0.46^***^	−0.29^**^	−0.14	−0.06	0.02	0.01	0.25^**^	−0.04	0.23^*^	0.20^*^	–	
13	Mental defeat (MD)	1.18 (0.98)	0.62^***^	0.54^***^	−0.28^***^		0.09	0.05	0.04	0.18	0.07	0.07	0.23^*^	0.64^***^	–
14	Negative self-related thoughts (PTCI)	2.58 (1.27)	0.39^***^	0.43^***^	−0.02	0.05	0.14	0.17	0.08	0.12	−0.02	0.09	0.16	0.32^***^	0.45^***^

PDS, Posttraumatic Diagnostic Scale; PSSI, Posttraumatic Symptom Severity Index; NART, National adult reading test; NEN, negative event narrative; TN, Trauma narrative; TPQ, Trauma processing questionnaire; MD, Mental Defeat scale; PTCI, Posttraumatic Cognition Inventory; Means and standard deviations for linguistic markers percentage scores (e.g., percentage of all words in a participant's narrative that were first person singular pronouns);

* p < 0.05,

** p < 0.005,

*** *p < 0.001*.

### Measures

#### PTSD Symptoms

PTSD symptom severity at 2 weeks was assessed using the self-report Post-traumatic Diagnostic Scale [PDS, (35); α =.92], symptom severity at 6 months was assessed with the PTSD Symptom Scale, a semi-structured interview with 17 items, each corresponding to one of the DSM-IV criteria for PTSD [PSSI, (36); interrater reliability, κ = 0.82]. We also indexed PTSD diagnosis using the PTSD Symptom Scale- Interview (PSS-I), a 17-item structured (36) and it was established that 18.9% of the sample fulfilled diagnostic criteria for PTSD at 6 months.

#### Verbal Intelligence

Participants also completed the National Adult Reading Test [NART, (37)], a widely accepted measure of verbal intelligence. The NART requires participants to read out loud a list of 50 irregularly spelled words in order of increasing difficulty. The number of words read correctly comprises the final score. The NART has excellent reliability and construct validity (38). It correlates highly with other measures of intelligence and allows the prediction of full-scale IQ scores (37).

#### Trauma-Specific and Non-specific Linguistic Markers

Verbatim transcripts of the narratives were analyzed using Linguistic Inquiry and Word Count [LIWC, (39)], an extensively validated computerized text-analysis tool. LIWC analyses texts by calculating the percentage of words in a given text that fall into a set of pre-defined psychological and grammatical categories. Based on prior research and cognitive PTSD theory, we limited our analysis to the categories *cognitive processes* (e.g., “cause,” “know,” “ought”) as an index of elaboration and cognitive processing, *death-related words* (e.g., “dead,” “kill,” “grave”) as an index of mental defeat and death salience, *negative emotions* (e.g., “angry,” “sad,” “cry”), as an index of negative emotionality, and first *person singular pronouns* (e.g., “I,” “me,” “my”) as an index of self-immersed processing. To address the question of process specificity, we computed separate word-use variables for the trauma and negative event narratives. Sample excerpts illustrating these four language variables in both types of narratives are provided in the Appendix.

#### Self-reported Peri- and Post-traumatic Processing

Data driven processing was assessed as an index of lacking elaboration and cognitive processing with the 8-items data-driven processing subscale from the Cognitive Processing Scale (40). The scale assesses the extent to which individuals engage in surface-level, perceptual processing during the assault (“I could not think clearly,” “I was confused and could not fully make sense of what was happening”), each on a scale from 0 (not at all) to 4 (very strongly). Internal consistency in the present sample was good, Cronbach's α = 0.87.

The Mental Defeat Scale (41), an 11-items self-report questionnaire, was used to index mental defeat. Participants rated the extent to which statements such as “I no longer felt like a human being” or “In my mind, I gave up” applied to them at some time during the assault, each on a scale from 0 (not at all) to 4 (very strongly). Internal consistency in the present sample was high, Cronbach's α = 0.90.

The Negative thoughts about the self subscale of the Post-traumatic cognition inventory [PTCI, (42)] was used to index self-related thoughts in context of the assault, each on a scale from 1 (totally agree) to 7 (totally disagree). The PTCI indexes generalized negative appraisals of the trauma and its aftermath and has been shown to have good reliability and convergent validity (42). Internal consistency of the 21-items negative self subscale in the present sample was high, Cronbach's α = 0.93.

### Data Analysis

We calculated a hierarchical linear regression analysis in order to determine whether trauma-specific linguistic markers predict PTSD symptom severity at 6 months, over and above initial PTSD symptoms, verbal intelligence and non-trauma linguistic variables. From the significant trauma-specific linguistic variables and corresponding self-report questionnaires, we determined the best set of variables to predict PTSD symptom severity using a stepwise linear regression and a forward selection method. All analyses were conducted using SPSS 20.0.

## Results

Results from the linear regression analyses are shown in Table 2. Less use of cognitive processing words in negative event narratives predicted PTSD symptom severity at 6 months after controlling for initial PTSD symptom severity and verbal intelligence. No other negative event linguistic characteristics predicted PTSD. However, when trauma-specific linguistic characteristics were introduced in the regression model, general cognitive processing from the negative event narrative was no longer significant. Instead, the linguistic trauma narrative characteristics, with exception of negative emotion words, significantly predicted later PTSD symptom severity. Less cognitive processing, use of more death-related words and more first person singular pronouns in trauma narratives were predictive of more PTSD symptoms at follow-up. Together, the linguistic predictors from the trauma narrative accounted for 7% additional variance in later PTSD symptoms, over and above initial PTSD symptoms, verbal intelligence and general linguistic predictors from non-trauma narratives.

**Table 2:** Trauma-specific linguistic markers predict posttraumatic stress disorder symptom severity at 6 months beyond initial symptom severity, verbal intelligence and non-specific linguistic markers.

	**Step Δ*R^2^***	**β**	***p***
Step 1^a^: Test of control variables	0.31^***^		<0.001
Initial PTSD symptom severity (PDS)		0.51	<0.001
Verbal intelligence (NART)		−0.18	0.012
Step 2^b^: Test of non-specific linguistic markers	0.04		0.119
Initial PTSD symptom severity (PDS)		0.55	<0.001
Verbal intelligence (NART)		−0.13	0.093
Cognitive processing NEN		−0.18	0.025
Death-related words NEN		0.06	0.437
Negative emotions NEN		−0.05	0.487
First person singular pronouns NEN		0.08	0.316
Step 3^c^: Test of trauma-specific linguistic markers	0.07^**^		0.007
Initial PTSD symptom severity (PDS)		0.50	<0.001
Verbal intelligence (NART)		−0.07	0.367
Cognitive processing NEN		−0.10	0.198
Death-related words NEN		0.04	0.617
Negative emotions NEN		−0.07	0.342
First person singular pronouns NEN		−0.03	0.728
Cognitive processing TN		−0.19	0.012
Death-related words TN		0.14	0.048
Negative emotions TN		0.06	0.365
First person singular pronouns TN		0.17	0.034

Dependent Variable = PTSD symptom severity at 6 months; PDS, Posttraumatic Diagnostic Scale; NART, National adult reading test; NEN, negative event narrative; TN, Trauma narrative; Predictor multicollinearity was within an acceptable range for all predictors, i.e., range of tolerance = 0.73–1.0.

** p < 0.005,

*** *p < 0.001*.

a *Step 1: R = 0.55, F_(2, 134)_ = 29.59, p < 0.001*.

b *Step 2: R = 0.59, F_(4, 130)_ = 11.37, p < 0.001*.

c *Step 3: R = 0.64, F_(4, 126)_ = 8.89, p < 0.001*.

In a separate model, we determined the best predictors of later PTSD symptom severity from the three significant trauma narrative linguistic variables (cognitive processing, use of death-related words, first person singular pronouns) and the corresponding self-report questionnaire scores (data-driven processing, mental defeat, and negative self-related thoughts) using linear regression and a forward selection procedure. First person singular pronouns in trauma narratives significantly predicted PTSD at 6 months, β = 0.17, *p* < 0.031, alongside self-reported mental defeat, β = −0.52, *p* < 0.001. No other significant predictor emerged in this analysis. Together, the variables explained 34% of PTSD symptom severity, *R* = 0.58, *F*_(1, 125)_ = 31.92, *p* < 0.001. There were no significant sex differences in magnitude in any of the linguistic predictors under study (all *p* > 122).

## Discussion

Linguistic markers of trauma-specific processing indexed in the early aftermath of a trauma uniquely predicted later chronic PTSD symptoms. Less cognitive processing words (as marker of less elaboration), use of more death-related words (as marker of mental defeat), use of more first person singular pronouns (as marker of self-immersed processing) assessed in the first 2 weeks after trauma predicted more severe PTSD symptoms at 6 months follow-up. Importantly, these linguistic predictors uniquely emerged from trauma-narrative competing against language markers of general emotional processing derived from non-traumatic, negative event narratives and predicted PTSD over and above established risk factors such as verbal intelligence and initial levels of PTSD symptoms.

The use of less cognition words in those with increased PTSD symptoms is in accord with cognitive theories of PTSD and the proposed beneficial nature of elaborative cognitive processing during trauma (13, 14) and complements earlier findings of increased cognition words predicting fewer distress symptoms following emotional events (15, 25). Although references to death were low overall, their relative frequencies predicted later PTSD, hence indicating the sensitivity of this index and its potential for clinical use. The finding is in line with studies showing perceived threat to life as an important aspect of trauma [for a review see (19)]. More reference to death and dying in trauma narratives may also reflect mental defeat and the result of giving up all efforts to retain one's identity as a human being during trauma. Negative emotion use in trauma narratives was, however, not predictive of later PTSD, a finding that replicates earlier results by Jones et al. (43) and may indicate limited usefulness of this index early post-trauma yet leaves open the possibility that PTSD-specific differences emerge gradually at a later time. Focus on specific emotional categories, such as shame or anger could be more predictive than a broad negative emotion category. Finally, more first-person singular pronoun use in trauma narratives predicted later PTSD and may indicate unmitigated self-immersion which may hamper emotion regulation and has been associated with greater impairment of mental and social functioning (9, 26).

Use of more first-person singular pronouns remained a significant predictor alongside self-reported mental defeat, when corresponding questionnaire measures were included. Assessment of such candidate early linguistic predictors, along with self-report questionnaires is thus one way to capture psychological processes after exposure to trauma and to predict later psychological adjustment. Self-report questionnaires may often provide practically the most efficient way of indexing psychological processing, despite their potential limitations when administered early after of trauma. However, patients routinely report on aspects of their trauma and such narratives could readily be subjected to automatic linguistic analyses as part of routine clinical practice.

The current study is not without limitations. Our sample consisted of assault survivors (who suffer a relatively high risk for chronic PTSD) and generalizability to other trauma types needs to be established. The potentially lower reliability of the negative event narrative markers due to their lower word count might have to some extent constrained their regression weights. Yet, LIWC variables are based on proportions and 50 or less words have proven sufficient to yield reliable estimates in prior research (44). Although, some of the narratives were rather short, minimum requirements for establishing LIWC scores were met for those narratives at the lower end of the word count. The results should nevertheless be replicated in another sample and specificity established, as well as reliability of the linguistic markers. Moreover, the linguistic predictors were conceptualized as proxies of psychological processes (e.g., mental defeat), which usually only partially convergence with questionnaire scores [see also (45)], owing, in part, also to methodological differences between these two methods of measurement. A fruitful approach could thus be to use both measures in complement to forecast trauma adaptation and development of later PTSD.

Despite these limitations, our findings have important clinical implications. We identified early linguistic markers of later PTSD assessed in narratives provided by survivors at 18 days post-trauma and found that they predicted 7% of later variance in PTSD over and above initial symptom severity. Self-immersed processing assessed by a linguistic index and mental defeat indexed by self-report questionnaire emerged as the best set of early predictors of later PTSD in the present study. Linguistic markers can thus make a clinically significant contribution and help identify those individuals at risk of developing chronic PTSD. Early provision of trauma-focused psychotherapy for these individuals at risk can prevent chronic PTSD (3). Linguistic markers, assessed early after trauma, may complement self-report questionnaires and help identify those at risk that could effectively be treated with such early psychological treatment, and they predicted over and above initial symptom severity. Reducing dysfunctional self-focus, self-immersed processing and putting the experience of mental defeat in perspective, as well as working through the trauma memory to enhance elaboration and cognitive processing comprise useful targets for such interventions.

## Ethics Statement

The protocol was approved by the IRB of King's College Hospital. All subjects gave written informed consent in accordance with the Declaration of Helsinki.

## Author Contributions

BK and AE developed the study concept and the study design. Testing and data collection were performed by BK. BK, AH, and AE performed the data analysis and interpretation. BK, RK, AH, and MM drafted the paper, and AE and MM provided critical revisions. All authors approved the final version of the paper for submission.

### Conflict of Interest Statement

The authors declare that the research was conducted in the absence of any commercial or financial relationships that could be construed as a potential conflict of interest.

## Footnotes

^1^Data are reported for those 136 participants who provided both narratives initially and who were reassessed at 6 months later. This subgroup did not differ from the total sample in terms of age, sex, or initial PTSD symptom severity.

^2^Other measures from the current sample are reported in (33, 34). None of these papers analyzed trauma or negative event narratives.
