# Supplementary material for: Early Linguistic Markers of Trauma-Specific Processing Predict Post-trauma Adjustment
Source: Front Psychiatry. 2018 Dec 5;9:645. doi: 10.3389/fpsyt.2018.00645 (PMC6290715; doi:10.3389/fpsyt.2018.00645)
Supplement: Supplementary file 1 [file Data_Sheet_1.docx]

Appendix

Sample excerpts with linguistic markers (in bold) for trauma and negative non-traumatic narratives

|  | Trauma narrative | Negative event narrative |
| --- | --- | --- |
| Cognitive processing | He basically came up to me and was standing with his face directly in front of mine and to be honest, I didn’t **think** he **would** even dream of like handling me. I was actually in that **belief** that he **would** not, he **would** not touch me. Err unfortunately I was wrong. | I **thought** it was all about something else, **because** the guy started getting very rude and very sort of, you know, err, uptight. So I got very aggressive, immediately, **because** I **assumed** that this was going to happen. |
| Death-related words | But I remember being scared, because I’m on the ground, and worried about getting **killed**, and worried about getting stomped to **death**. | I’ve been having problems sleeping lately, I stayed up and I was watching this film, a documentary about soldiers. Before a war there’s ninety-eight per cent of the soldiers, but when there’s a war, only two per cent do that, actual **killings** and that. |
| Negative emotions | At the time of the assault, I felt **frightened**, I felt **angry, confused**, roughly I don’t know what was happening to me. | I’m emotional, **devastated** and I’m **distressed**, because I can’t find anywhere to stay. |
| First person singular pronouns | **I** said “**I** want him arrested” and when **I** got in the ambulance **I** said “**I** don’t want him arrested”, because **I** love his mother. But **I** couldn’t take the beating, and then **I** had to think about the beating from the first time at this time and **I** cant’ cope, **I** can’t cope. **I** can’t sleep, **I** can’t eat. | And **I**’ve lost **my** things, **I**’ve lost **my** money, they took **my** money, they took **my** **my** pots, everything. **I**’ll have to get hold of **my** of the houses, **my** clothes. **I**’ve lost everything. |
